# Supplementary material for: Longitudinal study of calf morbidity and mortality on smallholder farms in southern Ethiopia
Source: PLoS One. 2021 Sep 16;16(9):e0257139. doi: 10.1371/journal.pone.0257139 (PMC8445444; doi:10.1371/journal.pone.0257139)
Supplement: Attachment — Submitted filename: Comments to Authors.docx [file pone.0257139.s003.docx]

Comments to author(s)

Thank you for submitting your research for consideration for publication with the PLOS ONE. Overall, I found this an interesting manuscript to read.

I have a made general comments followed by some specific comments.

***General comments:***

For future reference it would be useful to have line numbers at assist with reviewing. I have broken up the feedback into the manuscript headings and have either provide a line number or rewritten a sentence for the writer to consider.

Grammar throughout the manuscript requires addressing. I have addressed many of the issues throughout. However, it would be ideal to review this in detail.

References need to be in order as they appear throughout the paper.

Please check table numbers in text as they do not appear to match up with text. They need to appear in the manuscript in the order they are mentioned.

***Specific comments:***

**Title:**

“LONGITUDINAL STUDY ON CALF MORBIDITY AND MORTALITY IN MILK-SHED DISTRICTS OF SOUTHERN ETHIOPIA”

Please consider-wording as “milk-shed districts” is not the best description.

An example may be the following: “Longitudinal study of calf morbidity and mortality on smallholder dairy farms of southern Ethiopia”

**Key words**:

Remove “incidence”

**Background:**

Please change “This arises from death loss, treatment cost, decreased lifetime productivity and survivorship” to This arises from deaths, treatment cost, decreased lifetime productivity and longevity”

Please change “Likely, Smallholder dairy farmers experience high calf mortalities which can go up to 50% [29]” to “A study has shown that some smallholder dairy farmers may experience calf mortalities of up to 50% [29]”.

Pneumonia should not have a capital “P”

**Material and Methods:**

Study Area Description

Please change: “In these areas, about hundred smallholder households farming produce milk from cross bred and indigenous cattle breeds” to “In these districts, approximately one hundred smallholders produce milk from cross bred and indigenous cattle breeds”.

Please change : “According to the Gamo zone livestock and fishery resource department (2020), estimated livestock population of the zone is 1,297,173 cattle population.” To “According to the Gamo zone livestock and fishery resource department (2020), the estimated cattle population of the zone is 1,297,173.”

Please reference above in reference list

What is the refence for coordinates, rainfall etc? Please add this in and include in reference list.

Please change have a capital T for “the average annual temperature ranges from 22ºC to 28ºC and relative humidity is about 60% [18].”

Study Farms and Population

Please change “On the other hand, dairy farms located in the outskirts of the respective towns are considered as periurban dairy farms.” To “Dairy farms located on the outskirts of the respective towns are considered as periurban dairy farms.”

Please remove animals form the end of this sentence “Both Zebu and crossbred dairy calves of both sexes reared under smallholder dairy farms, calves aged between births to 6 months were considered in the study ~~animals~~.”

Please reword this sentence “However, calves born with stillbirth, anomaly and wasting diseases, as well as calves were not born in selected urbans and outskirts were ignored from this followup study upto accomplishing period”. Consider “Still born calves, calves with deformities, calves with a wasting diseases, as well as calves were not born in selected urbans and outskirts were excluded from this study.”

Please change “Following recruitment into the study, routine monitoring of study calves was done at 2 week intervals until 6 months old, morbidity or until death” to “Following recruitment into the study, routine monitoring of study calves was done at 2 week intervals until 6 months old”.

Study Design

Please change “ Cross sectional survey….” To “The cross sectional survey…..”

Please change “Questionnaire was administered to small-holder dairy producers during the study period for the herd-level data by in-depth interview aimed at soliciting information pertaining to on farm characteristics, calf and dam factors, management, health and environmental associated risk factors, and preparturient cow management practices. It used to support the prospective cohort results” to “The questionnaire was carried out by interview, to small-holder dairy producers during the study period to obtain herd-level data relating to farm characteristics, calf and dam factors, management, health and environmental associated risk factors, and pre-parturient cow management practices.”

Sampling technique and sample size determination

Line 7 please change “last” to late

Please complete this sentence “The total sample size for household interview was determined according to [6]”.

Line 12 remove randomly

Change “A prospective observational study that extended for approximately six months from starting of November, 2019 to April, 2020 was used to estimate the incidence of calf morbidity and mortality, and to assess the associated risk factors.” To “ A prospective observational study that conducted between November 2019 to April 2020, was used to estimate the incidence of calf morbidity and mortality, and to assess the associated risk factors.”

The following sentence is not clear. Please re-write/re-word “. In order to estimate calf morbidity and mortality and its potential risk factors among calves less than six months, as hypotheses have been proved that there is difference in incidence of morbidity and mortality between crossbred and local calves [30,36].”

Please change the following sentence from “A report of previous literature suggested that a birth to six months proportion of morbidity and mortality was 22.8% and 7.8% in crossbred and local calves respectively [36].” To “previous literature has shown that from birth to six months of age, the morbidity and mortality was 22.8% and 7.8%, in crossbred and local calves, respectively [36].”

Please change “Thus, 𝑛1 were 54 and 𝑛2 were 142 with total of 196, 54 local breed and 142 crossbred calves were sampled.” To “Thus, 𝑛1 was 54 and 𝑛2 was 142 with total of 196, 54 local breed and 142 crossbred calves were sampled.”

Farm visiting and calf recruitment criteria

Please change the following “Calves of less than 3 months of age at the initial visit with disease history and birth date known were recruited retrospectively and allowed to join into prospective cohort” to “Calves less than 3 months of age at the initial visit with a known date of birth and disease history were recruited retrospectively and allowed to join into prospective cohort”

Line 7- how regularly were the farms revisited? Study design need to be able to be repeated exactly the same by other investigators.

Please change “During each visit calves’ management, housing and sanitation situations were also observed.” To “ At each farm visit, calf management, housing and sanitation situations were also observed.

How were health event outside farm visit communicated to investigators? In writing, verbally etc?

Line 11- please remove from and replace with to

Please change “When the calf loss occurred during the follow up period, date and reason of loss were recorded.” To “When the calf loss occurred, date and reason of loss were recorded at the subsequent farm visit.”

Please change the following “When calves reached 6 months of age, they were withdrawn from the follow-up group.”

Please reword this sentence, as it is not clear to reader what you are trying to say “However, calves were identified as diseased or died before 6 months of age, was considered for the failure time.”

Data Management and Statistical Analysis

Please change “For this study purpose,” to “For the purpose of this study,”

Please change the following “Moreover, to facilitate result comparisons with other findings because of directly taking true rate results tend to overestimate calf morbidity and mortality rates [19], true rates calculated for mortality, morbidity and specific disease condition were derived into risk rates based on the formula (RR= 1-e -True Rate) [14].” To “Moreover, to facilitate result comparisons with other findings, true rates calculated for mortality, morbidity and specific disease condition were derived into risk rates based on the formula (RR= 1-e -True Rate) [14].”

Please change “ First, Kaplan–Meier method was employed to estimate hazard function of observed hazard differences for different explanatory variables with crude morbidity and mortality.” To “First, Kaplan–Meier method was employed to estimate hazard function of observed hazard differences for each of the explanatory variables with crude morbidity and mortality.”

Line 15: Please change “…to see their independent effect.” To “..to assess their independent effect.”

Please change the following “Finally, explanatory variables that best explained the calf morbidity and mortality were selected by a backward stepwise approach thereby from the model once a time explanatory variables that were not statistically significant were removed beginning with the least significant until the estimated regression coefficients for all retained variables were significant at (P ≤ 0.05). Thus, main effects were included in the final model.” To “Finally, explanatory variables that best explained the calf morbidity and mortality were selected by a backward stepwise selection. Starting with the least significant explanatory variable, each of the non-significant explanatory variable were removed until the estimated regression coefficients for all retained variables were significant (P ≤ 0.05). Thus, main effects were included in the final model.”

**RESULTS (should this heading be RESULTS and DISCUSSION? As there is interpretation of the results within this section. If not, then this needs to be edited to only provide results with no interpertation)**

Household characteristics of the respondents

Please remove “Livelihood”

There is no intext reference to Table 1- please provide intext reference or remove table one and re label all tables.

Please change the following “Among the total smallholder dairy producers interviewed in Gamo zone milk-shed districts, 65% in urban and 35% in peri-urban smallholder dairy farms (Table 3), and most (79.38%) of households were male headed and the rest (21.62%) were female headed (Figure 2). The household and dairy performance characteristics per small-holders farms are shown Table 2.” To “ Among the total smallholder dairy producers interviewed in Gamo zone milk-shed districts, 65% were urban and 35% were peri-urban smallholder dairy farms (Table 3), and 79.38% of households were male headed and 21.62% were female headed (Figure 2). The household and dairy performance characteristics per small-holders farms are shown Table 2.”

Please change the following from “As to respondents age, most (65%) of the respondents were under the age group of 41-80 years which is a mature age group (Figure 2).” To “In regards to respondents age, most (65%) of the respondents were in the age group of 41-80 years(Figure 2)”.

This sentence is discussion therefore needs to eb removed from the results section -“Urban and peri-urban dairy production is a recently growing livestock subsector and a means of family income and livelihood for some smallholders”

Farm management characteristics and calf health related problems

Please change “…in the districts are presented (Table 3)”. To “ “…in the districts are presented in Table 3.”

Line 3 remove “survey”

Line 6 change to the following “Majority (82%) calf husbandry practices were carried out by owner.”

This sentence needs to reworded as it is unclear “ In the study districts most of the calf caretakers (78.2%) carried out their work through longer work experience of greater than five years.” Do you mean “ In the study districts most of the calf caretakers (78.2%) had experience of greater than five years”.?

Please change the following sentence “Of the newborn calves, 66.8% were offered suckle and bucket feeding of colostrum within 1 to 6 hours after birth and all farmers allowed colostrum feeding immediately after the release of the placenta.” To “Of the newborn calves, 66.8% were allowed to suckle and/or bucket feed colostrum within 1 to 6 hours after birth”. ~~and all farmers allowed colostrum feeding immediately after the release of the placenta.~~

Prospective Cohort Study

Please change the following “During the start of study, calf with birth and history data were recruited concurrently were 45% of the total, and the remaining calves were resulted from births within the study time frame. Female and male calves attributed over the observation period were 108 (55%) and 88 (45%) respectively. These numbers of calves contributed a total of 28,043 and 32,012 calf-days, and 77.9 and 88.9 calf-years at risk for morbidity and mortality respectively (Table 4 and 5). “ to “ Forty-five percent of calves in the study had birth dates and history data at the commencement of the study, and the remaining recruited calves were born during the study period. A total of 108 (55%) female, and 88 (45%) male calves were enrolled in the study. These calves contributed a total of 28,043 and 32,012 calf-days, and 77.9 and 88.9 calf-years at risk for morbidity and mortality respectively (Table 4 and 5).”

Please change the following “The temporal pattern of the study cohort is shown (Table 3). Out of 196, 58 calves were exited due to diseases, deaths and sales from the study before termination of the study period. The total censor rate was 24.5% of which 24 and 34 were females and males, respectively. The censor rate due to deaths and sales was 13.3%. The reason for the majority exit account for males because small holders did not want male calves since they incur extra-feeding cost and relative cost in comparison with female calf was too cheap.” To “The temporal pattern of the study cohort is shown (Table 3). A total of 58 (24.5%) calves, 24 female and 34 male, were exited the study period due to diseases, deaths and sales. The censor rate due to deaths and sales was 13.3%. The majority of calves exiting were males calves that were sold because small holders did not want male calves since they incur extra-feeding cost and are less valuable then female calves.”

**Estimates of morbidity and mortality**

Incidence of morbidity

Line 2: Remove the brackets around Table 4.

Line 2-3: Replace follow up with follow-up

Line 3: Replace foremost with greatest

Reference Table 4 after this sentence “The risk rate for pneumonia, arthritis, navel illness and septicemic condition were all between 0.64% and 6.55%.” ie “The risk rate for pneumonia, arthritis, navel illness and septicemic condition were all between 0.64% and 6.55% (Table 4).”

Change the following “were 71.5, 66, 40 and 5.5 days respectively (Fig. 3).” To “were 71.5, 66, 40 and 5.5 days, respectively (Fig. 3).”

Please change the following “This finding indicated that calf diseases are the major problem at median age less than 3 months. Proportionally, the peak morbidity (10.4%) incidents occurred in the first week of life. Besides, 27% of the total cases of crude morbidity occurred in the first month of age and 72.9% of the total cases of crude morbidity occurred in the first three months of age..” To “ These finding indicated that disease commonly occurs in calves less than 3 months of age, with 10.7% morbidity occurring in the first week of life. Cumulatively, 27% morbidity occurred in the first month of life and 72.9% occurred in the first 3 months of life.”

Incidence of mortality

Remove the brackets around table 5.

Line 8, place comma before respectively

Please change the following “In this study, median survival time for crude mortality was 75 days under the age category of 1 - 4 months shown (Figure 4). As a result, calf age related health events can be grouped into younger group, which was the critical age for calf health. On the other hand, in the second group, the health events were less intense and declined gradually. Relatively higher risk of mortality in young calves age up to 4 months observed in this study was suggested the need of more careful management for very young calves as compared to older ones.”

To

“In this study, the median survival time for crude mortality was 75 days. This falls into the age category of 1 - 4 months shown in Figure 4. As a result, calf age related health events can be grouped into younger calves (<4 months), which is a critical age for calf health. In calves older than 4 months of age, the health events were less severe and declined gradually.

Relatively higher risk of mortality in young calves age up to 4 months observed in this study was suggested the need of more careful management for very young calves as compared to older ones.”

**Association of risk factors with incidence of morbidity and mortality**

Determinants of calf morbidity

Please change the following “…regression analysis of the breed..” to “…regression analysis for the breed…”

Please change the following “Essentially crossbred calves were got at higher risk than that of local breed ones.” To “Essentially crossbred calves were at greater risk of morbidity than that of local breed calves.”

Please change “The temporal distribution of calf breeds morbidity rate across six months of study period presented (Figure 5) were evidently indicated this association.” To “ The temporal distribution of calf breeds morbidity rate across six months of study period presented in Figure 5, demonstrates the above findings.”

Please change the following “The finding that delayed colostrum intake (> 6 hours of age) associated with hazard of morbidity.” To “The finding that delayed colostrum intake (> 6 hours of age) is associated with increased risk of morbidity”.

Replaced ultimate with increased

Please change “ Regarding to dam parity the….” To “In regards to dam parity, the…”

The following need to be removed as this is not an appropriate conclusion – “This might be due to the susceptibility of cows varies noticeably and new infections are most common in older cows at early lactation when the management was poor. Also, multiparous dam has high risk of clinical mastitis.”

There are several papers that discuss calf vigor and possible factors that influence calf vigor.

Determinants of crude mortality

Please change the following “When similar analysis to crude mortality was executed like crude morbidity, colostrum feeding adequacy, colostrum feeding status, time of colostrum ingestion, method of colostrum feeding and others were found with significant association (P ≤ 0.05) in the univariate Cox regression (Table 8).” To “Table 8 displays the significant (P ≤ 0.05) factors associated with morbidity in the univariate Cox regression.”

Please change the following “The difference in hazard of mortality between calves grouped by most significant factor, age at first colostrum feeding, was evidenced in the hazard function curve (Figure 7).” To “The difference in hazard of mortality between calves grouped by most significant factor, age at first colostrum feeding, was evident in the hazard function curve (Figure 7).”

Please change the following from “This finding showed higher hazard of the mortality in weaned calves below 4 months in comparison to above 4 months (P ˂ 0.05). Thus, in this study, calves stayed without being introduced to dry feed up to weaning age and no special starter feed requires special attention since weaned calves failed to adjust to dry feed quickly and capably that leads to malnutrition and susceptibility to diseases in semi-intensive farms. However, calves in mixed production usually start fresh grass before weaning.” To “This finding demonstrates a higher hazards of the mortality in weaned calves less than 4 months of age, when compared to calves greater than 4 months of age (P ˂ 0.05). In this study, it was common practice for calves to be not fed dry feed prior to weaning age. Therefore calves failed to adjust to dry feed quickly and capably that leads to malnutrition and susceptibility to diseases in semi-intensive farms when weaned. However, calves in mixed production usually start fresh grass before weaning and were more likely to remain healthy.”

**Discussion**

Please change “It has been thought that efficient dairy production and limited losses are important for farmers to realize maximum benefits from dairy segment” to “It has been thought that efficient dairy production and limited losses are important for farmers to achieve maximum productivity in dairy farming”.

Line 5: Replace fuller with greater

Please change the following “In urban and peri-urban vicinities of milk-shed districts of Gamo zone, the crude incidence risk of calf morbidity of 30.9% and calf mortality of 8.64% were recorded, and this record reflected moderate of the report so far in Ethiopia.” To “ Within the urban and peri-urban dairy facilities of the districts of Gamo zone, the crude incidence risk of calf morbidity of 30.9% and calf mortality of 8.64% were recorded. This greater than previous report in Ethiopia.

Please change the following “In the current study, the number of calves per farm was small and the smallholder dairy farmers so can easily monitor calves and take measures to avoid calf health problems. This could be explanation to discover comparatively lower mortality rate than those large herd size farms aforementioned. However, it is taken higher than that there 3 - 5% calf death that could be expected in good calf management and at economically tolerable level [13,21].” To “ In the current study, the number of calves per smallholder dairy farms was small, therefore each calf can be easily monitored and measures to avoid calf health problems. This could be an explanation as to why mortality rate should be less than those large herd size farms aforementioned. However, it is much higher than the economically tolerable level of 3 - 5% calf death[13,21].”

Please change the following “The incidence of crude morbidity recorded in this six months follow up study was 30.9%. This result is close to the findings of [10], where crude incidence rates of morbidity (29.3%). The result of the present study, however, is lower than the incidence rates of crude morbidity (62%) as reported by [39] and 66.7% morbidity as reported by [7] and 34.1% morbidity reported by [28].” To “ The incidence of crude morbidity recorded over the six months follow-up period was 30.9%. This result is close to the findings of Bekele et al, 2009[10], where crude incidence rates of morbidity (29.3%). The result in the present study, however, is lower than the incidence rates of crude morbidity (62%) as reported by Wudu et al 2008 [39] and 66.7% morbidity as reported by Assefa and Ashenafi 2016[7] and 34.1% morbidity reported by Mohammed et al 2020[28].”

Please change “This difference was might be due to management system, current better access to veterinary service in urban and suburbs and the owner of the calves, small herd size, good awareness about importance of colostrum and most of the owners feed colostrum to their calves immediately and this difference might be due to education level.” To “This difference might be due to better management system, better access to veterinary service in urban suburbs, small herd size, good awareness about importance of colostrum, most of the owners feed colostrum to their calves immediately. The latter difference might be due to education level.”

Please change “The incidence of mortality rate reported in this study was 8.64% with the range (5.4 - 13.7), to “The mortality rate reported in this study was 8.64% with the range (5.4 - 13.7)”.

Page 23 line 5 remove the words “respectively” and “lower”

Please reword this sentence as it is not clear “For instances, a calf mortality of 20.9% in the Oromia region [37], and greater than 50% in Tropics [29] who had been insisted better calf management practices.”

Please edit the following sentences as suggested:

The ~~incidence~~ risk of calf diarrhea was 10.43% in this study, which is comparable with the report of [10] (write author’s name and date) who documented 10% and [37] (write author’s name and date) who recorded 10.4%. In contrary, the present outcome was less than previous studies by [39] (write author’s name and date), 34%, (write author’s name and date) [24], 34.8% and (write author’s name and date) [7], 42.9%.

Please change the following “Similar result reports had been documented that such…” to Similar reports have documented that such…”

Please change the following “Likewise, the lower death losses due to mortality factors might be observed in farms where owner managed the calves than managed by hired labor performed husbandry [41].” To “Likewise, the lower death losses due to mortality factors might be observed in farms where calves are managed by farm owner rather than managed by hired labor[41].”

Please change the following “This finding has shown the importance of personal closeness between the owner and the calves that it might play key role for the animal welfare and health. This suggests that owners might be encouraged necessarily to provide the care necessary to assure high survival.” To “This finding has shown the importance of close relationship between the owner and the calves, and that this is likely to play a key role in improved animal welfare and health. ~~This suggests that owners might be encouraged necessarily to provide the care necessary to assure high survival.”.~~

Please change the following “It has been stated that early weaning is a risk factor for calf mortality statistically associated in Ethiopia [5,28,39] who found higher risk of mortality in calves that were weaned below 4 months as compared to those weaned at 4 or more months. In line with, this finding showed higher hazard of the mortality in weaned calves below 4 months.” To “It has been reported that early weaning is a risk factor for calf mortality in Ethiopia [5,28,39]. Calves have a higher risk of mortality when weaned below 4 months of age when compared to those weaned at 4 or greater than 4 months of age. The current study supported this finding.”

Please reword the following as it is unclear “However, the age reflected early and found out to be risky for calves in present study was optimum age for weaning in 23 the literatures found in Ethiopia [10,33]. Therefore, that observed in this follow up study was the actual weaning age for calves. Besides, calves stayed without being introduced to dry feed up to weaning age and no special starter feed was given in the study area.”

Please add in the author and date of paper in this sentence “This finding was in agreement with the finding of [39] who reported 2.24 times higher morbidity in calves ingested colostrum 6 hours later.”

Please change the following “This result is more or less similar with the reports by others shown in assessment of young stock mortality in different regions in Ethiopia [37].” To “This result is similar to the reports of young stock mortality in different regions in Ethiopia [37].”

Contrary to this result, other study, calves were fed partially or completely colostrum 1 to 2 hours without considering the release of placenta in India [32].

Please edit the following sentences as suggested: Please consider removing this release of placenta does not need be considered when it come to colostrum feeding.

“Contrary to this result, other study, calves were fed partially or completely colostrum 1 to 2 hours without considering the release of placenta in India [32]. “

**Conclusion**

Please change the following “Thus it was found higher than economically tolerable level” to “This level of morbidity and mortality is higher than economically tolerable for most smallholders.”

Please change the following “More significantly, smallholders kept smaller herd size and part of livelihood. Therefore, these higher rates of disease conditions and some extent of deaths was decreased replacement herd and ultimately hinder the success of smallholder dairy business. Calf diarrhea was the main calf health problem, whereas septicaemic conditions for deaths. The critical time for higher calf morbidity and mortality in this study was during the first 3 months of life up to the fourth month of age.” To “More significantly, smallholders have smaller herd sizes and the animals are a significant part of livelihood. Therefore, these higher rates of morbidity and some mortality decreased replacement herd numbers and ultimately hinder the success of smallholder dairy business. Calf diarrhea was the main calf health problem, whereas septicaemic conditions for deaths. The critical time for higher calf morbidity and mortality in this study was during the first 3 months of life.”

Please change the following “Moreover, study revealed malpractices…” to “ Moreover, this study revealed malpractices…”

Please change “However, most of host and management related determinants are responsive to intervention.” To “However, most of the host and calf caretakers are responsive to suggested intervention.”

Line 14: Remove strong

**Acknowledgements**

Please change the following “calves management system” to “dairy farms”.
