# Supplementary material for: Longitudinal study of calf morbidity and mortality on smallholder farms in southern Ethiopia
Source: PLoS One. 2021 Sep 16;16(9):e0257139. doi: 10.1371/journal.pone.0257139 (PMC8445444; doi:10.1371/journal.pone.0257139)
Supplement: Attachment — Submitted filename: Response to Reviewer comments.docx [file pone.0257139.s004.docx]

**Manuscript ID: PONE-D-21-01541R1**

**Title:** **"Longitudinal study of calf morbidity and mortality on smallholder farms in southern Ethiopia"**

**RESPONSE TO THE ACADAMIC EDITOR**

Comments to the Author

1. Please ensure that your manuscript meets PLOS ONE's style requirements

**Answer:** We regret for the error occurred by not following the PLOS ONE's style requirements and necessary corrections were made in the revised manuscript.

2. Significant text overlap between submission and the previously published works ((background, paragraph 1, sentence 2) (background, paragraph 2, sentences 1-2) (background, paragraph 3, sentence 1) (background, paragraph 3, sentence 3-6); (background, paragraph 7, sentence 8) (background, paragraph 8, sentence 1) (background, paragraph 8, sentences 4- 5) and (conclusion, paragraph 1, sentences 6-7))

**Answer:** We regret for the error occurred by the text overlap between our manuscript and previous published works and necessary corrections were made in the revised manuscript as follows which has highlighted by yellow colour.

- background, paragraph 1, **Page no:3/ line no: 65 – 67**
- background, paragraph 2, **Page no:3/ line no: 74 – 75**
- background, paragraph 3, **Page no:3/ line no: 80 – 82**
- background, paragraph 3, **Page no:3/ line no: 84 – 92**

3. Additional information inclusion

**Answer**: We have attached the necessary additional information that includes the questionnaire survey which is the herd-level cross-sectional study and the calf-level longitudinal study parts.

4. Minimal dataset have not specified that underline the results described in your manuscript

**Answer**: As we, authors, understood from the definition the minimal dataset for our manuscript includes certain parts of the values of behind hazard ratio, calf-survival days and herd-level survey responses and values we used to build graphs. Thus, we have attached as supporting information files.

5. We note Figure 1 in your submission contains map images which may be copyrighted.

**Answer**: We have used the study area map from maps produced by the Ethiopian cartography agency for country’s national data. However, we cannot obtain permission because it is already released to be used within the country. Thus, we used the second option and have removed the map from the manuscript.

6. PLOS requires an ORCID ID for the corresponding author

**Answer**: I have ORCID ID as per the PLOSE guideline for the corresponding author

7. The use of Table 1 that do not refer in the text of the manuscript

**Answer**: We regret for the error occurred by the use of Table 1 which is not appropriately depicted in the text of the manuscript and we have made necessary corrections in the revised manuscript (**Page no:8/ line no: 219**).

**RESPONSE TO THE REVIEWER’S COMMENTS**

**Reviewer #1: General comments**

1. I have a made general comments followed by some specific comments

**Answer**: We, authors, are grateful for the reviewer’s suggestions on this manuscript. The authors thank the reviewer for recommending this manuscript to be proceed for possible publication and ready for necessary corrections

2. For future reference it would be useful to have line numbers at assist with reviewing.

**Answer**: We have included the line numbers as it has seen in revised manuscript

3. Grammar throughout the manuscript requires addressing

**Answer**: We have addressed all the grammar errors and were made necessary corrections

4. References need to be in order as they appear throughout the paper

**Answer**: Author regret for the disorderly put references and necessary corrections were made in the revised manuscript.

5. Table numbers do not appeared to match up with text

**Answer**: Author regret for the error and necessary corrections were made in the revised manuscript

**Reviewer #1: Specific comments**

6. Title modification

**Answer**: Author accept title modification and necessary corrections were made

**From** “LONGITUDINAL STUDY ON CALF MORBIDITY AND MORTALITY IN MILK-SHED DISTRICTS OF SOUTHERN ETHIOPIA**” to “**Longitudinal study of calf morbidity and mortality on smallholder dairy farms of southern Ethiopia**” (Page no:1/ line no: 1 – 2)**

7. Key words: Remove “incidence”

**Answer**: We accepted and removed all key words including the “Incidence” according to the PLOSOne_formatting_sample_main_body (**Page no:2/ line no: 52 – 53**)

8. Please change “This arises from death loss, treatment cost, decreased lifetime productivity and survivorship” to “This arises from deaths, treatment cost, decreased lifetime productivity and longevity”

**Answer**: Changed to “This arises from deaths, treatment cost, decreased lifetime productivity and longevity” (**Page no:2/ line no: 74 – 75**)

9. Please change “Likely, Smallholder dairy farmers experience high calf mortalities which can go up to 50%” to “A study has shown that some smallholder dairy farmers may experience calf mortalities of up to 50%”

**Answer**: Removed from manuscript completely

10. First letter heading of “Pneumonia”

**Answer**: Removed from manuscript completely due to modification

11. Study Area Description
12. Change “In these areas, about hundred smallholder households farming produce milk from cross bred and indigenous cattle breeds” to “In these districts, approximately one hundred smallholders produce milk from cross bred and indigenous cattle breeds”
13. Answer: Changed to “In these districts, approximately one hundred smallholders produce milk from cross bred and indigenous cattle breeds” (**Page no:4/ line no: 99 – 100**)
14. Change “According to the Gamo zone livestock and fishery resource department (2020), estimated livestock population of the zone is 1,297,173 cattle population.” To “According to the Gamo zone livestock and fishery resource department (2020), the estimated cattle population of the zone is 1,297,173.”

**Answer**: Changed to “According to the Gamo zone livestock and fishery resource department (2020), the estimated cattle population of the zone is 1,297,173” (**Page no:4/ line no: 102 – 103**) and have included “Reference” and has corrected spelling errors (**Page no:4/ line no: 117**)

15. Change “On the other hand, dairy farms located in the outskirts of the respective towns are considered as periurban dairy farms.” To “Dairy farms located on the outskirts of the respective towns are considered as periurban dairy farms.”

**Answer**: Changed to “Dairy farms located on the outskirts of the respective towns are considered as periurban dairy farms” (**Page no:5/ line no: 122 – 123**)

16. Remove the word “animals” from the end of this sentence

**Answer**: Removed “Both Zebu and crossbred dairy calves of both sexes reared under smallholder dairy farms, calves aged between births to 6 months were considered in the study” (**Page no:5/ line no: 124 – 125**)

17. Reword this sentence “However, calves born with stillbirth, anomaly and wasting diseases, as well as calves were not born in selected urbans and outskirts were ignored from this follow up study upto accomplishing period”

**Answer**: The sentence was paraphrased “However, still born calves, calves with deformities and wasting diseases, as well as calves that were not born in selected urbans and outskirts were excluded from this study.” (**Page no:5/ line no: 127 – 128**)

18. Change “Following recruitment into the study, routine monitoring of study calves was done at 2 week intervals until 6 months old, morbidity or until death” to “Following recruitment into the study, routine monitoring of study calves was done at 2 week intervals until 6 months old”

**Answer**: Changed to “Following recruitment into the study, routine monitoring of study calves was done at 2 week intervals until 6 months old” (**Page no:5/ line no: 128 – 129**)

19. Change “Cross sectional survey….” To “The cross sectional survey…..”

**Answer**: Changed to “The cross sectional survey” (**Page no:5/ line no: 132**)

20. Change “Questionnaire was administered to small-holder dairy producers during the study period for the herd-level data by in-depth interview aimed at soliciting information pertaining to on farm characteristics, calf and dam factors, management, health and environmental associated risk factors, and preparturient cow management practices. It used to support the prospective cohort results” to “The questionnaire was carried out by interview, to small-holder dairy producers during the study period to obtain herd-level data relating to farm characteristics, calf and dam factors, management, health and environmental associated risk factors, and pre-parturient cow management practices.”

**Answer**: Changed to “The questionnaire was carried out by interview, to small-holder dairy producers during the study period to obtain herd-level data relating to farm characteristics, calf and dam factors, management, health and environmental associated risk factors, and pre-parturient cow management practices” (**Page no:5/ line no: 134 - 138**)

21. Change “last” to “late”

**Answer**: Changed to “late” (**Page no:5/ line no: 144**)

22. Complete this sentence “The total sample size for household interview was determined according to [6]”

**Answer**: Modified the sentence to “The total sample size for household interview was determined according to Arsham [6].” (**Page no:5/ line no: 147**)

23. Remove the word “randomly” from the end of this sentence

**Answer**: The word “randomly” do not removed because calves were included into study by random sampling technique (**Page no:5/ line no: 151**)

24. Change “A prospective observational study that extended for approximately six months from starting of November, 2019 to April, 2020 was used to estimate the incidence of calf morbidity and mortality, and to assess the associated risk factors.” To “A prospective observational study that conducted between November 2019 to April 2020, was used to estimate the incidence of calf morbidity and mortality, and to assess the associated risk factors.”

**Answer**: Changed to “A prospective observational study that conducted between November 2019 to April 2020, was used to estimate the incidence of calf morbidity and mortality, and to assess the associated risk factors.” (**Page no:5/ line no: 152 – 154**)

25. This sentence is not clear. Please re-write/re-word “. In order to estimate calf morbidity and mortality and its potential risk factors among calves less than six months, as hypotheses have been proved that there is difference in incidence of morbidity and mortality between crossbred and local calves [30,36].”

**Answer**: Modified for clarity to “Different works of literature have been proved that there is a difference in the incidence of morbidity and mortality between crossbred and local calves [30,36]” (**Page no:5/ line no: 155 – 157**)

26. Change this sentence from “A report of previous literature suggested that a birth to six months proportion of morbidity and mortality was 22.8% and 7.8% in crossbred and local calves respectively [36].” To “previous literature has shown that from birth to six months of age, the morbidity and mortality was 22.8% and 7.8%, in crossbred and local calves, respectively [36].”

**Answer**: Changed to “previous literature has shown that from birth to six months of age, the morbidity and mortality was 22.8% and 7.8%, in crossbred and local calves, respectively [36].” (**Page no:6/ line no: 157 – 158**)

27. Change “Thus, 𝑛1 were 54 and 𝑛2 were 142 with total of 196, 54 local breed and 142 crossbred calves were sampled.” To “Thus, 𝑛1 was 54 and 𝑛2 was 142 with total of 196, 54 local breed and 142 crossbred calves were sampled.”

**Answer**: Changed to “Thus, 𝑛1 was 54 and 𝑛2 was 142 with total of 196, 54 local breed and 142 crossbred calves were sampled.” (**Page no:6/ line no: 170 – 171**)

28. Change “Calves of less than 3 months of age at the initial visit with disease history and birth date known were recruited retrospectively and allowed to join into prospective cohort” to “Calves less than 3 months of age at the initial visit with a known date of birth and disease history were recruited retrospectively and allowed to join into prospective cohort”

**Answer**: Changed to “Calves less than 3 months of age at the initial visit with a known date of birth and disease history were recruited retrospectively and allowed to join into prospective cohort” (**Page no:6/ line no: 174 – 176**)

29. How regularly were the farms revisited? Study design need to be able to be repeated exactly the same by other investigators.

**Answer**: Following recruitment into the study, routine monitoring of study calves was done at 2 week intervals until 6 months old by authors (**Page no:5/ line no: 128 – 130**). Besides, authors assigned enumerator until the calves reached 6 months of age (**Page no:6/ line no: 180**)

30. Change “During each visit calves’ management, housing and sanitation situations were also observed.” To “At each farm visit, calf management, housing and sanitation situations were also observed.”

**Answer**: Changed to “At each farm visit, calf management, housing and sanitation situations were also observed” (**Page no:6/ line no: 181**)

31. How were health event outside farm visit communicated to investigators?

**Answer**: The farm and its outside health condition and management during our regular visit was communicated by farm owners and assigned enumerators. In addition, investigators made direct observation on the status of farm and its surrounding. The whole farm health status was good we meant that regular farm waste removal was experienced by the farm. However, the outside of farm has no biosecurity. This may be related to small number of farm size and smallholders did not consider it. The owners always strictly observe the farm’s hygiene and make every effort to avoid bringing disease onto the farm. They don’t mix up animals from other farmers and join the new comer animals.

When new cases appeared they communicate to local veterinary service delivery office and get service. During the study period, they call to assigned data enumerator and directly to the investigator when any health problem in the farm occurs.

32. Please remove from and replace with “to”.

**Answer**: It was corrected (**Page no:6/ line no: 184**)

33. Change “When the calf loss occurred during the follow up period, date and reason of loss were recorded.” To “When the calf loss occurred, date and reason of loss were recorded at the subsequent farm visit.”

**Answer**: Changed to “When the calf loss occurred, date and reason of loss were recorded at the subsequent farm visit.” (**Page no:6/ line no: 185 - 186**)

34. Change “When calves complete their 6 months of age, they were withdrawn from the follow-up group.” To “When calves reached 6 months of age, they were withdrawn from the follow-up group.”

**Answer**: Changed to “When calves reached 6 months of age, they were withdrawn from the follow-up group.” (**Page no:7/ line no: 187**)

35. It is not clear to reader what you are trying to say “However, calves were identified as diseased or died before 6 months of age, was considered for the failure time.”

**Answer**: It was modified to have clarity “calves that diseased or died before 6 months of age were considered for the failure time.” (**Page no:7/ line no: 188**)

36. Change “For this study purpose,” to “For the purpose of this study,”

**Answer**: Changed to “For the purpose of this study,” (**Page no:7/ line no: 193**)

37. Change “Moreover, to facilitate result comparisons with other findings because of directly taking true rate results tend to overestimate calf morbidity and mortality rates [19], true rates calculated for mortality, morbidity and specific disease condition were derived into risk rates based on the formula (RR= 1-e ^-True Rate^) [14]” To “Moreover, to facilitate result comparisons with other findings, true rates calculated for mortality, morbidity and specific disease condition were derived into risk rates based on the formula (RR= 1-e -True Rate) [14].”

**Answer**: Changed to “Moreover, to facilitate result comparisons with other findings, true rates calculated for mortality, morbidity and specific disease condition were derived into risk rates based on the formula (RR= 1-e -True Rate) [14].” (**Page no:7/ line no: 194 - 196**)

38. Change “First, Kaplan–Meier method was employed to estimate hazard function of observed hazard differences for different explanatory variables with crude morbidity and mortality.” To “First, Kaplan–Meier method was employed to estimate hazard function of observed hazard differences for each of the explanatory variables with crude morbidity and mortality.”

**Answer**: Changed to “First, Kaplan–Meier method was employed to estimate hazard function of observed hazard differences for each of the explanatory variables with crude morbidity and mortality.” (**Page no:7/ line no: 198 - 199**)

39. Change…to see their independent effect.” To “…. to assess their independent effect.”

**Answer**: Changed to “…. to assess their independent effect” (**Page no:7/ line no: 206**)

40. Change “Finally, explanatory variables that best explained the calf morbidity and mortality were selected by a backward stepwise approach thereby from the model once a time explanatory variables that were not statistically significant were removed beginning with the least significant until the estimated regression coefficients for all retained variables were significant at (P ≤ 0.05). Thus, main effects were included in the final model.” To “Finally, explanatory variables that best explained the calf morbidity and mortality were selected by a backward stepwise selection. Starting with the least significant explanatory variable, each of the non-significant explanatory variable were removed until the estimated regression coefficients for all retained variables were significant (P ≤ 0.05). Thus, main effects were included in the final model.”
41. **Answer**: Changed to “Finally, explanatory variables that best explained the calf morbidity and mortality were selected by a backward stepwise selection. Starting with the least significant explanatory variable, each of the non-significant explanatory variable were removed until the estimated regression coefficients for all retained variables were significant (P ≤ 0.05).” (**Page no:7/ line no: 206 - 2010**)
42. RESULTS (should this heading be RESULTS and DISCUSSION?)

**Answer:** The heading should only be RESULTS. We regret for the error included certain interpretations in the result section. Thus, corrections were made in the revised manuscript

43. Remove “Livelihood”

**Answer**: The word “Livelihood” was removed from the sentence (**Page no:8/ line no: 219**)

44. There is no in text reference to Table 1- please provide in text reference or remove table one and re label all tables.

**Answer**: We regret for the error and necessary corrections were made in the revised manuscript (**Page no:8/ line no: 219 – 220**)

45. Change “Among the total smallholder dairy producers interviewed in Gamo zone milk-shed districts, 65% in urban and 35% in peri-urban smallholder dairy farms (Table 3), and most (79.38%) of households were male headed and the rest (21.62%) were female headed (Figure 2). The household and dairy performance characteristics per small-holders farms are shown Table 2.” To “ Among the total smallholder dairy producers interviewed in Gamo zone milk-shed districts, 65% were urban and 35% were peri-urban smallholder dairy farms (Table 3), and 79.38% of households were male headed and 21.62% were female headed (Figure 2). The household and dairy performance characteristics per small-holders farms are shown Table 2.”

**Answer**: Changed to “Among the total smallholder dairy producers interviewed in Gamo zone milk-shed districts, 65% were urban and 35% were peri-urban smallholder dairy farms, and 79.38% of households were male headed and 21.62% were female headed (Figure 1)” (**Page no:8/ line no: 221 - 224**)

46. Change “As to respondents age, most (65%) of the respondents were under the age group of 41-80 years which is a mature age group (Figure 2).” To “In regards to respondents age, most (65%) of the respondents were in the age group of 41-80 years (Figure 2)”.

**Answer**: Changed to “In regards to respondents age, most (65%) of the respondents were in the age group of 41-80 years (Figure 2)” (**Page no: 9/ line no: 239 - 240**)

47. Change “…in the districts are presented (Table 3)”. To “…in the districts are presented in Table 3.”

**Answer**: Changed to “…in the districts are presented in Table 2.” Authors, regret for the error in text Table number and corrected in revised manuscript (**Page no: 9/ line no: 246**)

48. Remove “survey”

**Answer**: The word “survey” was removed from the sentence (**Page no:9/ line no: 246**)

49. Change “About 82% of calf husbandry practices…” to “Majority (82%) calf husbandry.…”

**Answer**: Changed to “Majority (82%) calf husbandry practices were carried out by owner.”

50. This reword sentence “In the study districts most of the calf caretakers (78.2%) carried out their work through longer work experience of greater than five years.”

**Answer**: modified to have clear meaning to “In the study districts most of the calf caretakers (78.2%) had experience of greater than five years” “Yes!” (**Page no:9/ line no: 250 - 251**)

51. Change “Of the newborn calves, 66.8% were offered suckle and bucket feeding of colostrum within 1 to 6 hours after birth and all farmers allowed colostrum feeding immediately after the release of the placenta.” To “Of the newborn calves, 66.8% were allowed to suckle and/or bucket feed colostrum within 1 to 6 hours after birth”

**Answer**: Changed to “Of the newborn calves, 66.8% were allowed to suckle and/or bucket feed colostrum within 1 to 6 hours after birth” (**Page no:9/ line no: 255 - 256**)

52. Change “During the start of study, calf with birth and history data were recruited concurrently were 45% of the total, and the remaining calves were resulted from births within the study time frame. Female and male calves attributed over the observation period were 108 (55%) and 88 (45%) respectively. These numbers of calves contributed a total of 28,043 and 32,012 calf-days, and 77.9 and 88.9 calf-years at risk for morbidity and mortality respectively (Table 4 and 5).” to “ Forty-five percent of calves in the study had birth dates and history data at the commencement of the study, and the remaining recruited calves were born during the study period. A total of 108 (55%) female, and 88 (45%) male calves were enrolled in the study. These calves contributed a total of 28,043 and 32,012 calf-days, and 77.9 and 88.9 calf-years at risk for morbidity and mortality respectively (Table 4 and 5).”

**Answer**: Changed to “Forty-five percent of calves in the study had birth dates and history data at the commencement of the study, and the remaining recruited calves were born during the study period. A total of 108 (55%) female and 88 (45%) male calves were enrolled in the study. These calves contributed a total of 28,043 and 32,012 calf-days, and 77.9 and 88.9 calf-years at risk for morbidity and mortality respectively (Table 4 and 5).” (**Page no: 10/ line no: 274 - 279**)

53. Change “The temporal pattern of the study cohort is shown (Table 3). Out of 196, 58 calves were exited due to diseases, deaths and sales from the study before termination of the study period. The total censor rate was 24.5% of which 24 and 34 were females and males, respectively. The censor rate due to deaths and sales was 13.3%. The reason for the majority exit account for males because small holders did not want male calves since they incur extra-feeding cost and relative cost in comparison with female calf was too cheap.” To “The temporal pattern of the study cohort is shown (Table 3). A total of 58 (24.5%) calves, 24 female and 34 male, were exited the study period due to diseases, deaths and sales. The censor rate due to deaths and sales was 13.3%. The majority of calves exiting were males calves that were sold because small holders did not want male calves since they incur extra-feeding cost and are less valuable then female calves.”

**Answer**: Changed to “The temporal pattern of the study cohort is shown (Table 3). A total of 58 (24.5%) calves, 24 female and 34 male, were exited the study period due to diseases, deaths and sales. The censor rate due to deaths and sales was 13.3%. The majority of calves exiting were male calves that were sold because small holders did not want male calves since they incur extra-feeding cost and are less valuable then female calves.” (**Page no: 11/ line no: 280 - 284**)

54. Reference Table 4 after this sentence “The risk rate for pneumonia, arthritis, navel illness and septicemic condition were all between 0.64% and 6.55%.” ie “The risk rate for pneumonia, arthritis, navel illness and septicemic condition were all between 0.64% and 6.55% (Table 4).”

**Answer**: Modified accordingly “The risk rate for pneumonia, arthritis, navel illness and septicemic condition were all between 0.64% and 6.55% (Table 4).”

55. Change “were 71.5, 66, 40 and 5.5 days respectively (Fig. 3).” To “were 71.5, 66, 40 and 5.5 days, respectively (Fig. 3).”

**Answer**: Changed to “were 71.5, 66, 40 and 5.5 days, respectively (Fig. 2).” (**Page no: 11/ line no: 299**). Here, the Figure No. has changed because the study area map was removed.

56. Change “This finding indicated that calf diseases are the major problem at median age less than 3 months. Proportionally, the peak morbidity (10.4%) incidents occurred in the first week of life. Besides, 27% of the total cases of crude morbidity occurred in the first month of age and 72.9% of the total cases of crude morbidity occurred in the first three months of age.” To “These finding indicated that disease commonly occurs in calves less than 3 months of age, with 10.7% morbidity occurring in the first week of life. Cumulatively, 27% morbidity occurred in the first month of life and 72.9% occurred in the first 3 months of life.”

**Answer**: Changed to “These finding indicated that disease commonly occurs in calves less than 3 months of age, with 10.7% morbidity occurring in the first week of life. Cumulatively, 27% morbidity occurred in the first month of life and 72.9% occurred in the first 3 months of life.” (**Page no: 12/ line no: 300 – 302**).

57. Remove the brackets around table 5 and place comma before respectively

**Answer**: It was corrected (**Page no: 13/ line no: 306 & 311**)

58. Change “In this study, median survival time for crude mortality was 75 days under the age category of 1 - 4 months shown (Figure 4). As a result, calf age related health events can be grouped into younger group, which was the critical age for calf health. On the other hand, in the second group, the health events were less intense and declined gradually. Relatively higher risk of mortality in young calves age up to 4 months observed in this study was suggested the need of more careful management for very young calves as compared to older ones.”

**Answer**: Changed to “In this study, the median survival time for crude mortality was 75 days. This falls into the age category of 1 - 4 months shown in Figure 3. As a result, calf age related health events can be grouped into younger calves (<4 months), which is a critical age for calf health. In calves older than 4 months of age, the health events were less severe and declined gradually. Relatively higher risk of mortality in young calves age up to 4 months observed in this study was suggested the need of more careful management for very young calves as compared to older ones.” (**Page no: 14/ line no: 319 – 325**).

59. Change “…regression analysis of the breed….” to “…regression analysis for the breed…”

**Answer**: Changed to “…regression analysis for the breed….” (**Page no: 15/ line no: 330**).

60. Change “Essentially crossbred calves were got at higher risk than that of local breed ones.” To “Essentially crossbred calves were at greater risk of morbidity than that of local breed calves.”

**Answer**: Changed to “Essentially crossbred calves were at greater risk of morbidity than that of local breed calves.” (**Page no: 15/ line no: 331 – 332**).

61. Change “The temporal distribution of calf breeds morbidity rate across six months of study period presented (Figure 5) were evidently indicated this association.” To “ The temporal distribution of calf breeds morbidity rate across six months of study period presented in Figure 5, demonstrates the above findings.”

**Answer**: Changed to “The temporal distribution of calf breeds morbidity rate across six months of study period presented in Figure 5, demonstrates the above findings.” (**Page no: 15/ line no: 332 – 333**).

62. Change “The finding that delayed colostrum intake (> 6 hours of age) associated with hazard of morbidity.” To “The finding that delayed colostrum intake (> 6 hours of age) is associated with increased risk of morbidity”

**Answer**: Changed to “The finding that delayed colostrum intake (> 6 hours of age) is associated with increased risk of morbidity.” (**Page no: 17/ line no: 350 – 351**).

63. Change “Regarding to dam parity the….” To “In regards to dam parity, the…”
    **Answer**: Changed to “In regards to dam parity, the……. (**Page no: 17/ line no: 355**).
64. Change “When similar analysis to crude mortality was executed like crude morbidity, colostrum feeding adequacy, colostrum feeding status, time of colostrum ingestion, method of colostrum feeding and others were found with significant association (P ≤ 0.05) in the univariate Cox regression (Table 8).” To “Table 8 displays the significant (P ≤ 0.05) factors associated with morbidity in the univariate Cox regression.”

**Answer**: Changed to “Table 8 displays the significant (P ≤ 0.05) factors associated with morbidity in the univariate Cox regression.” (**Page no: 18/ line no: 363 – 364**).

65. Change “The difference in hazard of mortality between calves grouped by most significant factor, age at first colostrum feeding, was evidenced in the hazard function curve (Figure 7).” To “The difference in hazard of mortality between calves grouped by most significant factor, age at first colostrum feeding, was evident in the hazard function curve (Figure 7).”

**Answer**: Changed to “The difference in hazard of mortality between calves grouped by most significant factor, age at first colostrum feeding, was evident in the hazard function curve (Figure 6).” (**Page no: 18/ line no: 377 – 379**).

66. Change “This finding showed higher hazard of the mortality in weaned calves below 4 months in comparison to above 4 months (P ˂ 0.05). Thus, in this study, calves stayed without being introduced to dry feed up to weaning age and no special starter feed requires special attention since weaned calves failed to adjust to dry feed quickly and capably that leads to malnutrition and susceptibility to diseases in semi-intensive farms. However, calves in mixed production usually start fresh grass before weaning.” To “This finding demonstrates a higher hazards of the mortality in weaned calves less than 4 months of age, when compared to calves greater than 4 months of age (P ˂ 0.05). In this study, it was common practice for calves to be not fed dry feed prior to weaning age. Therefore calves failed to adjust to dry feed quickly and capably that leads to malnutrition and susceptibility to diseases in semi-intensive farms when weaned. However, calves in mixed production usually start fresh grass before weaning and were more likely to remain healthy.”

**Answer**: Changed to “This finding demonstrates a higher hazard of the mortality in weaned calves less than 4 months of age, when compared to calves greater than 4 months of age (P ˂ 0.05). In this study, it was common practice for calves to be not fed dry feed prior to weaning age. Therefore calves failed to adjust to dry feed quickly and capably that leads to malnutrition and susceptibility to diseases in semi-intensive farms when weaned. However, calves in mixed production usually start fresh grass before weaning and were more likely to remain healthy.” (**Page no: 19/ line no: 382 – 388**).

67. Change “It has been thought that efficient dairy production and limited losses are important for farmers to realize maximum benefits from dairy segment” to “It has been thought that efficient dairy production and limited losses are important for farmers to achieve maximum productivity in dairy farming”.

**Answer**: Changed to “It has been thought that efficient dairy production and limited losses are important for farmers to achieve maximum productivity in dairy farming.” (**Page no: 20/ line no: 396 – 397**).

68. Replace fuller with greater

**Answer**: Replaced by “greater” (**Page no: 20/ line no: 400**).

69. Change “In urban and peri-urban vicinities of milk-shed districts of Gamo zone, the crude incidence risk of calf morbidity of 30.9% and calf mortality of 8.64% were recorded, and this record reflected moderate of the report so far in Ethiopia.” To “Within the urban and peri-urban dairy facilities of the districts of Gamo zone, the crude incidence risk of calf morbidity of 30.9% and calf mortality of 8.64% were recorded. This greater than previous report in Ethiopia.”

**Answer**: Changed to “Within the urban and peri-urban dairy facilities of the districts of Gamo zone, the crude incidence risk of calf morbidity of 30.9% and calf mortality of 8.64% were recorded. This is relatively lower than previous report in Ethiopia.” (**Page no: 20/ line no: 405 – 407**). Besides, we, authors, regret for the error comparison made and necessary corrections were made in the revised manuscript.

70. Change “In the current study, the number of calves per farm was small and the smallholder dairy farmers so can easily monitor calves and take measures to avoid calf health problems. This could be explanation to discover comparatively lower mortality rate than those large herd size farms aforementioned. However, it is taken higher than that there 3 - 5% calf death that could be expected in good calf management and at economically tolerable level [13,21].” To “In the current study, the number of calves per smallholder dairy farms was small, therefore each calf can be easily monitored and measures to avoid calf health problems. This could be an explanation as to why mortality rate should be less than those large herd size farms aforementioned. However, it is much higher than the economically tolerable level of 3 - 5% calf death [13,21].”

**Answer**: Changed to “In the current study, the number of calves per smallholder dairy farms was small; therefore, each calf can be easily monitored and measures to avoid calf health problems. This could be an explanation as to why mortality rate should be less than those large herd size farms aforementioned. Thus, it is much higher than the economically tolerable level of 3 - 5% calf death [13,21]” (**Page no: 20/ line no: 411 – 415**).

71. Change “The incidence of crude morbidity recorded in this six months follow up study was 30.9%. This result is close to the findings of [10], where crude incidence rates of morbidity (29.3%). The result of the present study, however, is lower than the incidence rates of crude morbidity (62%) as reported by [39] and 66.7% morbidity as reported by [7] and 34.1% morbidity reported by [28].” To “The incidence of crude morbidity recorded over the six months follow-up period was 30.9%. This result is close to the findings of Bekele et al, 2009[10], where crude incidence rates of morbidity (29.3%). The result in the present study, however, is lower than the incidence rates of crude morbidity (62%) as reported by Wudu et al 2008 [39] and 66.7% morbidity as reported by Assefa and Ashenafi 2016 [7] and 34.1% morbidity reported by Mohammed et al 2020 [28].”
72. **Answer**: Changed to “The incidence of crude morbidity recorded over the six months follow-up period was 30.9%. This result is close to the findings of Bekele et al, 2009[10], where crude incidence rates of morbidity (29.3%). The result in the present study, however, is lower than the incidence rates of crude morbidity (62%) as reported by Wudu et al 2008 [39] and 66.7% morbidity as reported by Assefa and Ashenafi 2016 [7] and 34.1% morbidity reported by Mohammed et al 2020 [28].” (**Page no: 20/ line no: 417 – 422**).
73. Change “This difference was might be due to management system, current better access to veterinary service in urban and suburbs and the owner of the calves, small herd size, good awareness about importance of colostrum and most of the owners feed colostrum to their calves immediately and this difference might be due to education level.” To “This difference might be due to better management system, better access to veterinary service in urban suburbs, small herd size, good awareness about importance of colostrum, most of the owners feed colostrum to their calves immediately. The latter difference might be due to education level.”

**Answer**: Changed to “This difference might be due to better management system, better access to veterinary service in urban and its suburbs, small herd size, good awareness about importance of colostrum, most of the owners feed colostrum to their calves immediately. The latter difference might be due to education level.” (**Page no: 20/ line no: 422 – 425**).

74. Change “The incidence of mortality rate reported in this study was 8.64% with the range (5.4 - 13.7), to “The mortality rate reported in this study was 8.64% with the range (5.4 - 13.7)”.

**Answer**: Changed to “The mortality rate reported in this study was 8.64% with the range (5.4 - 13.7)” (**Page no: 21/ line no: 429**).

75. Remove the words “respectively” and “lower”

**Answer**: the words “respectively” and “lower” has not removed because (1) authors used “respectively” for two studies subsequently put and used “lower” there is comparison between studies (**Page no: 21/ line no: 432 & 433**).

76. Reword this sentence as it is not clear “For instances, a calf mortality of 20.9% in the Oromia region [37], and greater than 50% in Tropics [29] who had been insisted better calf management practices.”

**Answer**: It was modified to have clarity as “For instances, calf mortality of 20.9% in the Oromia region [37], and calf mortality of greater than 50% in Tropics [29] who had been insisted better calf management practices.” (**Page no: 21/ line no: 434 – 435**).

77. Modify “The incidence risk of calf diarrhea was 10.43% in this study, which is comparable with the report of [10] (write author’s name and date) who documented 10% and [37] (write author’s name and date) who recorded 10.4%. In contrary, the present outcome was less than previous studies by [39] (write author’s name and date), 34%, (write author’s name and date) [24], 34.8% and (write author’s name and date) [7], 42.9%.”

**Answer**: It was modified to have clarity as “The risk of calf diarrhea was 10.43% in this study, which is comparable with the report of Bekele *et al*. [10] who reported 10%, Tsegaw *et al*. [37] who recorded 10.4%. In contrary, the present outcome was less than previous studies by Wudu *et al*. [39], 34%, Islam *et al*. [24], 34.8% and Assefa and Ashenafi, [7], 42.9%.” (**Page no: 21/ line no: 437 – 440**).

78. Change “Similar result reports had been documented that such…” to similar reports have documented that such…”

**Answer**: Changed to “….to similar reports have documented that such….” (**Page no: 21/ line no: 450**).

79. Change “Likewise, the lower death losses due to mortality factors might be observed in farms where owner managed the calves than managed by hired labor performed husbandry [41].” To “Likewise, the lower death losses due to mortality factors might be observed in farms where calves are managed by farm owner rather than managed by hired labor [41].”

**Answer**: Changed to “Likewise, the lower death losses due to mortality factors might be observed in farms where calves are managed by farm owner rather than managed by hired labor [41].” (**Page no: 21/ line no: 452 – 453**).

80. Change “This finding has shown the importance of personal closeness between the owner and the calves that it might play key role for the animal welfare and health. This suggests that owners might be encouraged necessarily to provide the care necessary to assure high survival.” To “This finding has shown the importance of close relationship between the owner and the calves, and that this is likely to play a key role in improved animal welfare and health.”

**Answer**: Changed to “This finding has shown the importance of close relationship between the owner and the calves, and that this is likely to play a key role in improved animal welfare and health.” (**Page no: 21/ line no: 453 – 455**).

81. Change “It has been stated that early weaning is a risk factor for calf mortality statistically associated in Ethiopia [5,28,39] who found higher risk of mortality in calves that were weaned below 4 months as compared to those weaned at 4 or more months. In line with, this finding showed higher hazard of the mortality in weaned calves below 4 months.” To “It has been reported that early weaning is a risk factor for calf mortality in Ethiopia [5,28,39]. Calves have a higher risk of mortality when weaned below 4 months of age when compared to those weaned at 4 or greater than 4 months of age. The current study supported this finding.”

**Answer**: Changed to “It has been reported that early weaning is a risk factor for calf mortality in Ethiopia [5,28,39]. Calves have a higher risk of mortality when weaned below 4 months of age when compared to those weaned at 4 or greater than 4 months of age. The current study supported this finding.” (**Page no: 22/ line no: 456 – 458**).

82. Reword this sentence as it is unclear “However, the age reflected early and found out to be risky for calves in present study was optimum age for weaning in 23 the literatures found in Ethiopia [10,33]. Therefore, that observed in this follow up study was the actual weaning age for calves. Besides, calves stayed without being introduced to dry feed up to weaning age and no special starter feed was given in the study area.”

**Answer**: Modified to be clear as “However, the age supposed early and found to be risk factor for calves in present study was optimum age for weaning in the literatures in Ethiopia [10,33]. Besides, calves stayed without being introduced to dry feed up to weaning age and no special starter feed was given in the study area.” (**Page no: 22/ line no: 459 – 462**).

83. Add in the author and date of paper in this sentence “This finding was in agreement with the finding of [39] who reported 2.24 times higher morbidity in calves ingested colostrum 6 hours later.”

**Answer**: Modified to be clear as “This finding was in agreement with the finding of Wudu *et al*. [39] who reported 2.24 times higher morbidity in calves ingested colostrum 6 hours later.” (**Page no: 22/ line no: 470 – 472**).

84. Change “This result is more or less similar with the reports by others shown in assessment of young stock mortality in different regions in Ethiopia [37].” to “This result is similar to the reports of young stock mortality in different regions in Ethiopia [37].”

**Answer**: Modified to be clear as “This result is similar to the reports of young stock mortality in different regions in Ethiopia [37].” (**Page no: 22/ line no: 480 – 481**).

85. Modify “Contrary to this result, other study, calves were fed partially or completely colostrum 1 to 2 hours without considering the release of placenta in India [32].”

**Answer**: Modified to be clear as “Contrary to this result, other study, calves were fed partially or completely colostrum 1 to 2 hours without considering the release of placenta in India [32].” (**Page no: 22/ line no: 481 – 483**). Besides, in Ethiopia farmers await for release of placenta to be released before letting the calves to suckle or bucket feeding. Thus, we tried its comparison with India.

86. Change “Thus it was found higher than economically tolerable level” to “This level of morbidity and mortality is higher than economically tolerable for most smallholders.”

**Answer**: Modified to be clear as “This level of morbidity and mortality is higher than economically tolerable for most smallholders.” (**Page no: 23/ line no: 493 – 494**).

87. Change “More significantly, smallholders kept smaller herd size and part of livelihood. Therefore, these higher rates of disease conditions and some extent of deaths was decreased replacement…...” To “More significantly, smallholders have smaller herd sizes and the animals are a significant part of livelihood. Therefore, these higher rates of morbidity and some mortality decreased replacement herd numbers and ultimately hinder the success of smallholder dairy business. Calf diarrhea was the main calf health problem, whereas septicaemic conditions for deaths. The critical time for higher calf morbidity and mortality in this study was during the first 3 months of life.”

**Answer**: Modified to be clear as “More significantly, smallholders have smaller herd sizes and the animals are a significant part of livelihood. Therefore, these higher rates of morbidity and some mortality decreased replacement herd numbers and ultimately hinder the success of smallholder dairy business. Calf diarrhea was the main calf health problem, whereas septicaemic conditions for deaths. The critical time for higher calf morbidity and mortality in this study was during the first 3 months of life.” (**Page no: 23/ line no: 494 – 499**).

88. Change “However, most of host and management related determinants are responsive to intervention.” To “However, most of the host and calf caretakers are responsive to suggested intervention.”

**Answer**: Changed to “However, most of the host and calf caretakers are responsive to suggested intervention.” (**Page no: 23/ line no: 502 – 503**).

89. Change “calves management system” to “dairy farms”.

**Answer**: Changed to “dairy farms.” (**Page no: 23/ line no: 508**).

**Reviewer #2: General comments**

1. Overall it is an interesting work on calf morbidity and mortality in countries like Ethiopia where dairy production is unused and on its verge of growing.

**Answer**: We, authors, are grateful for the reviewer’s suggestions on this manuscript. The authors thank the reviewer for recommending this manuscript to be preceded for possible publication and ready for necessary corrections.

2. The paper needs revision before considering it for publication.

**Answer**: We, authors, regret for the error and necessary corrections were made in the revised manuscript.

3. Grammatical flaws and English language proficiency needs to be given due consideration

**Answer**: Grammatical flaws and English language proficiency related issues’ necessary corrections were made in the revised manuscript.

**Reviewer #2: Specific comments**

4. Description of the diary production characteristics of the country

**Answer**: It was included and corrections were made in the revised manuscript (**Page no: 3/ line no: 62 – 64 & Page no: 4/ line no: 121 – 122**).

5. In last paragraph of the introduction on lines 4-6, mortality proportions but claimed as “rates”, which needs correction

**Answer**: Corrections were made in the revised manuscript (**Page no: 3/ line no: 79 – 84** ).

6. Strong justification is not provided

**Answer**: Justification was given in the revised manuscript (**Page no: 3/ line no: 88 – 94**).

7. The descriptions of the dairy farms used are not given

**Answer**: It was given and corrections were made in the revised manuscript (**Page no: 4/ line no: 119 – 125**).

8. Regarding misspelling

**Answer**: Words like small-holders and cross-sectional study was corrected **(Page no: 3/ line no: 119 & 124 & Page no: 5/ line no: 132 etc**).

9. Regarding study designs

**Answer**: We developed two study designs for data collection. Hence, the cross-sectional the design was used to collect the general herd-level data by interviewing the respondents about past events in the snapshot, whereas the longitudinal design was used for monitoring the incidence of morbidity and mortality about six months period. Thus, we believe the aim of both study designs and collected data were different and those variables can be done differently under respective study designs.

10. Regarding the family size

**Answer**: In Table 1, the total family size presented was 984 which means that the total number of individuals under the family of smallholders who were interviewed for this study (N = 160).

In the Table 2, the chi-squared test compared the proportion of respondents in each category of the variables studied to the calf health and mortality problems in farms for the past 1-year event occurrence as shown in the questionnaire (N=52). Thus, we suppose these variables categories have association with calf morbidity and mortality, and they may become determinant to the outcome of interest. By the way, N = 52 is the number of calf health and mortality problems faced in the past 1year in the respective study farms.

11. Regarding lost-to-follow-up population

**Answer**: Of course, 29.59% of the original study population was lost. However, the study population is an open cohort and replacement of the population was made particularly for withdrawal, hence 24.5% of calves were exited in the study period due to diseases, deaths, and sales. The study focus was animal-time at-risk, so-called incidence rate, but not cumulative incidence. However, according to Martin *et al*. (1987) we can convert the incidence rate (True Rate) values to the cumulative incidence whether the cohort is open or closed but not neglecting actual measure of association.

Open population: incidence risk = 1−exp(-incidence rate x length of study period). This formula is used to relate prevalence and incidence

We recorded all the outcomes and most explanatory variables from direct observations and suppose the test for association with disease outcome, however it might need cautious considerations due to the small number of death events which lower the power of the test in survival analysis.

12. Regarding the use of discussion with proportions (prevalence)

**Answer**: The discussion using proportions have made based on the conversion of the incidence rate (true rate) to cumulative incidence (risk rate) according to Martin *et al*. (1987) and Stevenson (2005) for open population computation of prevalence. With this regard the objective of this study was not only incidence rate but also cumulative incidence, because the objective as indicated in the last paragraph introduction section was designed to estimate the incidence of calf morbidity and mortality and the associated risk factors (**Page no: 3/ line no: 92 – 94**).

**Reference**

Stevenson, M. (2005). An Introduction to Veterinary Epidemiology. EpiCentre, IVABS

Massey University, Palmerston North, New Zealand
